# Cleavage of poly(A)-binding protein by duck hepatitis A virus 3C protease

**DOI:** 10.1038/s41598-017-16484-1

**Published:** 2017-11-24

**Authors:** Di Sun, Mingshu Wang, Xingjian Wen, Anchun Cheng, Renyong Jia, Kunfeng Sun, Qiao Yang, Ying Wu, Dekang Zhu, Shun Chen, Mafeng Liu, Xinxin Zhao, Xiaoyue Chen

**Affiliations:** 1 Institute of Preventive Veterinary Medicine, Sichuan Agricultural University, Wenjiang, Chengdu, Sichuan 611130 P.R. China; 2 Key Laboratory of Animal Disease and Human Health of Sichuan Province, Sichuan Agricultural University, Wenjiang, Chengdu, Sichuan 611130 P.R. China; 3 Avian Disease Research Center, College of Veterinary Medicine, Sichuan Agricultural University, Wenjiang, Chengdu, Sichuan 611130 P.R. China

## Abstract

During viral infections, some viruses subvert the host proteins to promote the translation or RNA replication with their protease-mediated cleavage. Poly (A)-binding protein (PABP) is a target for several RNA viruses; however, the impact of duck hepatitis A virus (DHAV) on PABP remains unknown. In this study, we demonstrated for the first time that DHAV infection stimulates a decrease in endogenous PABP and generates two cleavage fragments. On the basis of *in vitro* cleavage assays, an accumulation of PABP cleavage fragments was detected in duck embryo fibroblast (DEF) cell extracts incubated with functional DHAV 3C protease. In addition, DHAV 3C protease was sufficient for the cleavage of recombinant PABP without the assistance of other eukaryotic cellular cofactors. Furthermore, using site-directed mutagenesis, our data demonstrated a 3C protease cleavage site located between Q367 and G368 in duck PABP. Moreover, the knockdown of PABP inhibited the production of viral RNA, and the C-terminal domain of PABP caused a reduction in viral replication compared to the N-terminal domain. Taken together, these findings suggested that DHAV 3C protease mediates the cleavage of PABP, which may be a strategy to manipulate viral replication.

## Introduction

In eukaryotes, the 5′-terminal cap structure is necessary to initiate translation. During translation initiation, the m7G (5′) ppp (5′) N structure is first recognized by translation eukaryotic initiation factor 4 F (eIF4F). The eIF4F complex consists of the eIF4G scaffolding protein, the eIF4A RNA helicase and the eIF4E cap-binding protein. The 43S complex, containing eukaryotic initiation factors (eIFs) and the ternary complex (eIF2-GTP-Met-tRNA) together with the 40S ribosomal subunit, is recruited to the 5′-cap structure. The mRNA is activated by the binding of eIF4F to the cap and the binding of the poly(A) tail to poly(A)-binding protein (PABP). Then, the 43S ribosome scans down the 5′UTR to recognize AUG^1^. The interactions between PABP and several factors, including eIF4G, eIF4B, PCBP2 (poly r(C)-binding protein 2) and Paip (PABP-interacting protein), are necessary to stimulate translation. Of these interactions, PABP-eIF4G is best understood, while the eIF4B and PCBP2 binding sites on PABP remain unclear^2–5^. Hence, PABP (also known as cytoplasmic PABPC1) plays key roles in cellular gene expression.

Diverse viruses have developed various strategies to compete for the host translation machinery due to their limited genetic information. Picornaviruses utilize an internal ribosome entry site (IRES) to initiate viral translation instead of a 7-methylguanosine cap^6^. Other genetic elements in picornaviruses play significant roles in viral biology, for example, the removal of poly(A) blocks the infectivity of poliovirus (PV)^7^. Moreover, the viral cloverleaf structure is required for nucleic acid stability, translation, and replication of the virus. In addition, IRES recruits the 40S ribosomal subunit, and the cleavage of PABP is required for the switch from IRES-driven translation to RNA replication^8,9^. Above all, PABP is a significant regulatory protein in the translation processes of both cells and picornaviruses.

Picornavirus-encoded proteases include L protease, 2A protease and 3C protease, which cleave host translational factors such as eIF4A I, eIF4G I, and eIF5B to inhibit the cap-dependent translation of the host cell^10,11^. However, these proteases in different picornaviruses exhibit varying effects on the same cellular protein. For example, enterovirus 2A protease and aphthovirus L protease cleave eIF4G, whereas eIF4G seems to not be a substrate for 3C protease in hepatitis A virus (HAV). For duck hepatitis A virus (DHAV), intact eIF4G was reported to be essential for the internal initiation of translation^12^. As another central regulator, the multifunctional PABP is a target for RNA viruses. Although PABP is cleaved by 3C protease in infection with PV, HAV, encephalomyocarditis virus (EMCV) and foot-and-mouth disease virus (FMDV), the recognition sites in PABP vary somewhat^9,13,14^. Meanwhile, the relationship between PABP and DHAV is completely unknown.

DHAV is a highly fatal infectious disease in young ducklings and causes significant economic losses to the duck industry worldwide^15–19^. DHAV belongs to the *Avihepatovirus* genus in the *Picornaviridae* family. The picornaviral 5′UTR and 3′UTR are necessary for efficient translation. Reportedly, the DHAV IRES is distinct from type IV IRESs, although it does share common features with type IV IRES elements of picornaviruses^12^. However, the translation of DHAV might not be modulated by its 3′UTR sequence^20^. In addition, there are three structurally different 2A proteins in the polyprotein of DHAV^21^, including an aphthovirus-like 2A1^22^, a conserved avrRpt2-induced gene (AIGI) protein 2A2^21^, and a parechovirus-like 2A3^23^. Hence, 3C protease is the only viral protease in the DHAV polyprotein. In a previous study, DHAV IRES-directed translation was insensitive to eIF4G cleavage by FMDV L protease, while cap-dependent expression was inhibited^24^. However, the DHAV IRES activity was abolished with the addition of 2A protease from swine vesicular disease virus (SVDV), a member of the *Enterovirus* genus^12^. Although proteolysis of PABP was observed in cells infected with FMDV, L protease was confirmed to cleave both eIF4GI and eIF4GII but not PABP^25–28^. Remarkably, 2A protease of PV could cleave eIF4G and PABP^29–31^. Due to these similarities and differences between DHAV and other picornaviruses, more studies are needed. Here, we analysed for the first time the interaction between DHAV 3C protease and duck PABP *in vitro* and *in vivo* to determine the function of 3C protease in the viral life cycle.

## Results

### Endogenous PABP is modified during DHAV infection

**Figure 1:**
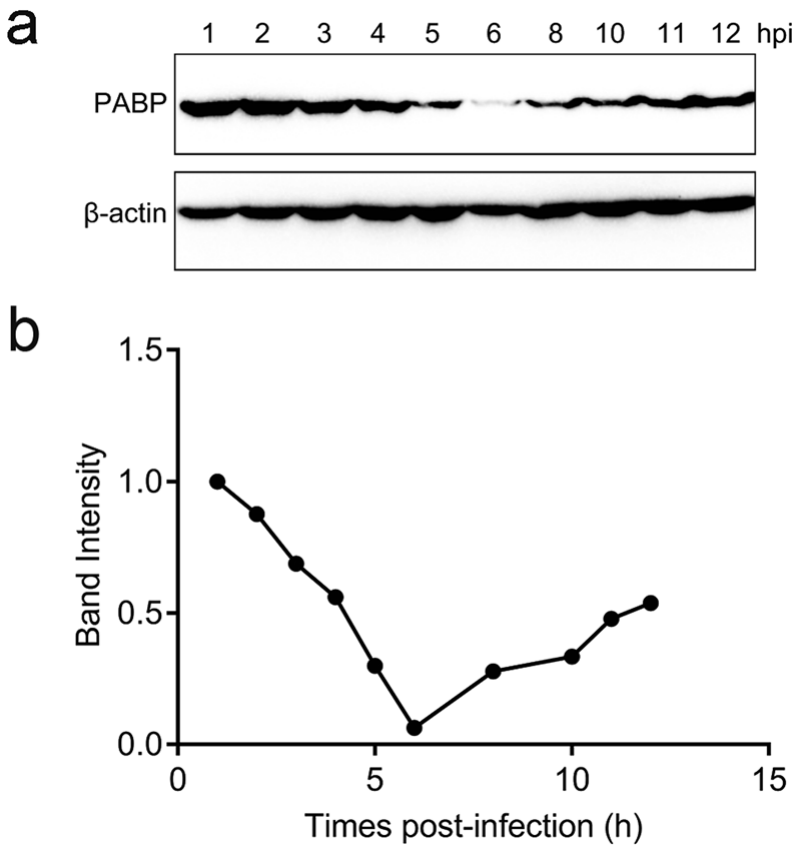
Change in cellular poly(A)-binding protein (PABP) during the duck hepatitis A virus (DHAV) infection of duck embryo fibroblast (DEF) cells. (**a**) Western blotting analysis was performed to detect the protein expression levels of PABP, with β-actin as the loading control. (**b**) Band intensity of PABP in the DHAV infection. The band intensities representing PABP protein expression levels were quantitated using the β-actin control bands as a reference (for each time point) using the Image J software.

**Figure 2:**
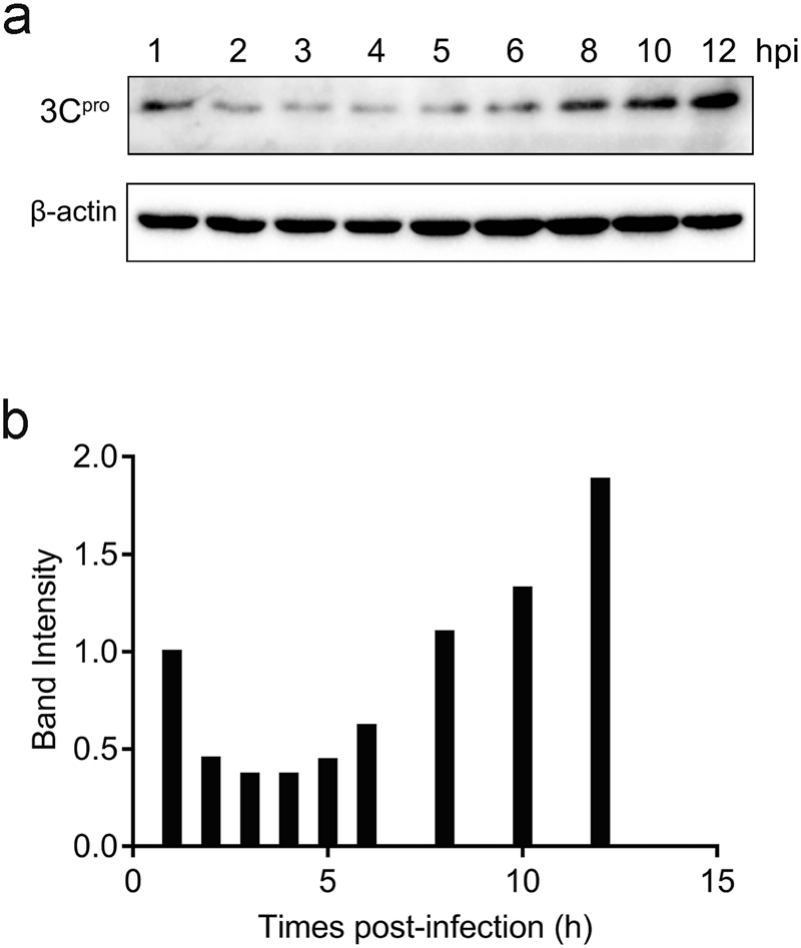
Accumulation of viral 3C protease in DHAV-infected cells. (**a**) Time course study of DHAV 3C protease expression via immunoblotting. In addition, β-actin was used as a control. (**b**) Band intensity of 3C protease during DHAV infection. The band intensities representing the 3C protease level were quantitated using the β-actin control bands as a reference.

In this study, experiments were performed in duck embryo fibroblast (DEF) cells to investigate whether there were any detectable changes in native PABP or any other detectable changes during DHAV infection, as translational control pathways have been reported to be changed or dysregulated in some established cell lines^32^. To determine the influence of DHAV infection on endogenous PABP, DEF cells were infected with DHAV (1000 TCID_50_) and collected with cell lysis buffer at various times post-infection. The samples from each time point were fractionated by sodium dodecyl sulfate polyacrylamide gel electrophoresis (SDS-PAGE) and then analysed by immunoblotting with anti-PABP and anti-3C serum. The relative ratios of PABP were calculated compared with the housekeeping enzyme β-actin at each time point. Before 2 hpi, the 70 kDa band corresponding to the full-length PABP was relatively stable. However, the decrease in the PABP levels was first detected at approximately 3 hpi. After 8 hpi, the relative PABP levels increased gradually (Fig. 1a and b). The expression level of 3C protease increased over time in DEF cells infected with DHAV, while the β-actin levels remained relatively stable during infection (Fig. 2a and b). The decrease in PABP was most likely due to viral proteolytic cleavage, as observed in infection with other picornaviruses. During DHAV infection, the PABP cleavage products were not detected along with the change in PABP. The anti-PABP serum (10E10) is reportedly unable to detect the cleavage products of PABP generated by enterovirus proteases. This monoclonal anti-PABP serum was tested for interaction with PABPs from various species by Western blotting in a previous study and here was confirmed to recognize duck PABP but not only human, rabbit or chicken PABP^33^.

### DHAV 3C protease and PABP co-localization during DHAV infection

**Figure 3:**
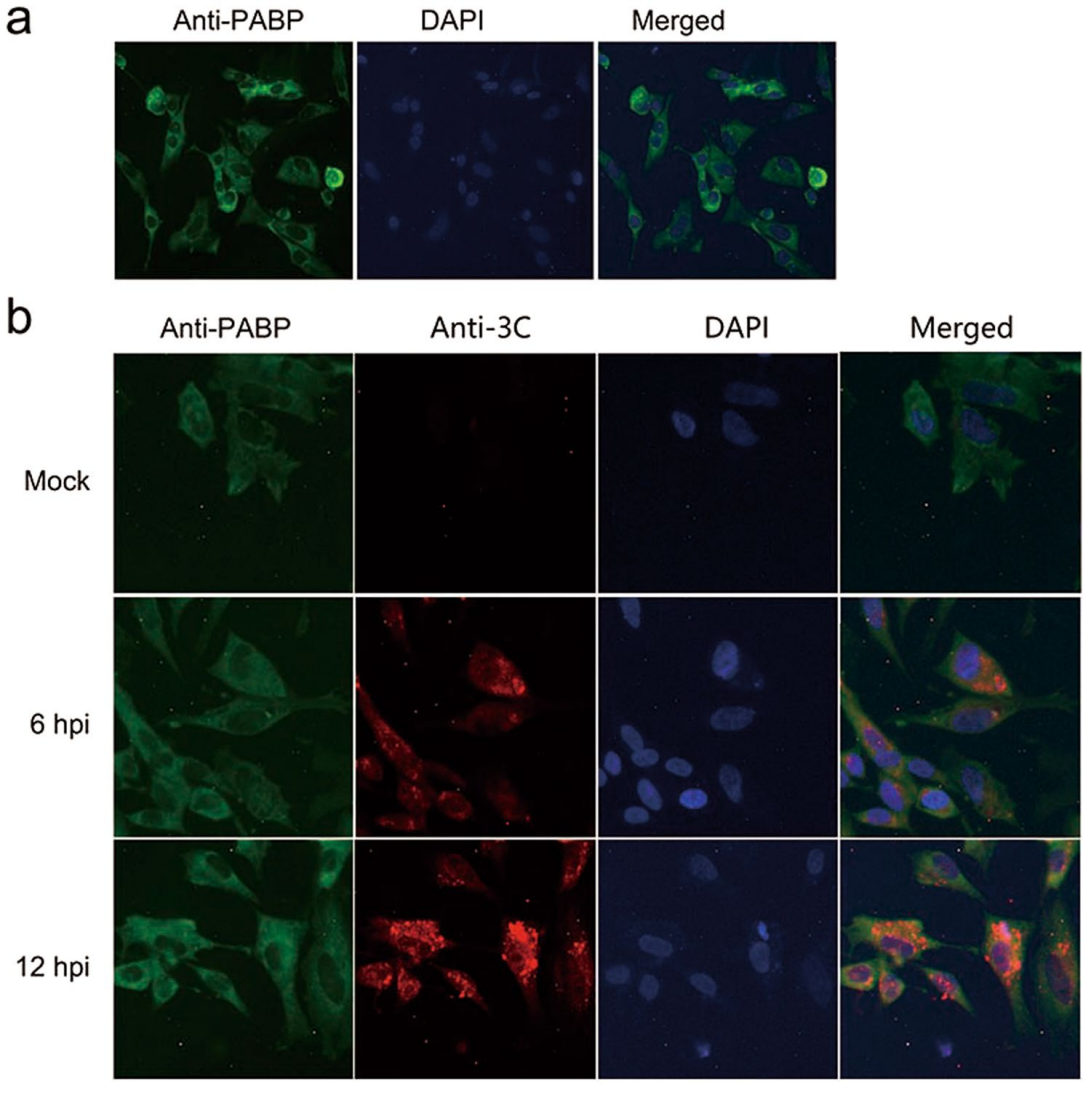
DHAV 3C protease and PABP co-localization during DHAV infection. (**a**) The localization of PABP (green) in DEF cells. DAPI staining shows the nucleus in blue. (**b**) The DEF cells were mock treated or infected with DHAV for 6 h and 12 h. The localization of 3C protease (red) and PABP (green) was detected using indirect immunofluorescence (IF).

Interactions between viral proteins and cellular proteins are required for multiple steps in the infection process, including the cleavage of these cellular proteins and the blocking of their function. The anti-PABP serum was used to examine the cellular distribution of PABP in DEF cells. Indirect immunofluorescence (IF) showed that duck PABP was localized throughout the cytoplasm and did not accumulate in the nucleus of DEF cells (Fig. 3a). Likewise, human PABP was found to be localized exclusively in the cytoplasm of HeLa cells and not in the nucleus^33^. These results suggested that anti-PABP (10E10) could recognize duck PABP. Then, the relative localization of PABP and 3C protease in cells mock treated or infected with DHAV were determined. DEF cells infected with DHAV were processed for IF at the indicated times post-infection. At 6 hpi, PABP was localized throughout the cytoplasm. Meanwhile, 3C protease was detected in the cytoplasm surrounding the nucleus and in small amounts in the nucleus. In contrast, at 12 hpi, the 3C protease-specific fluorescence signal was clearly stronger and showed translocation into the nucleus. No significant red fluorescence was observed in mock-infected cells (Fig. 3b). This result was consistent with the previous finding that PV 3C protease was initially localized in the cytoplasm and then translocated into the nucleus within 2–4 h of infection^34^. It has been reported that 3C protease can regulate the replication of the host cell in the nucleus. Our results suggest that DHAV 3C protease can enter the nucleus.

### Specific cleavage of PABP by DHAV 3C protease in uninfected cells

**Figure 4:**
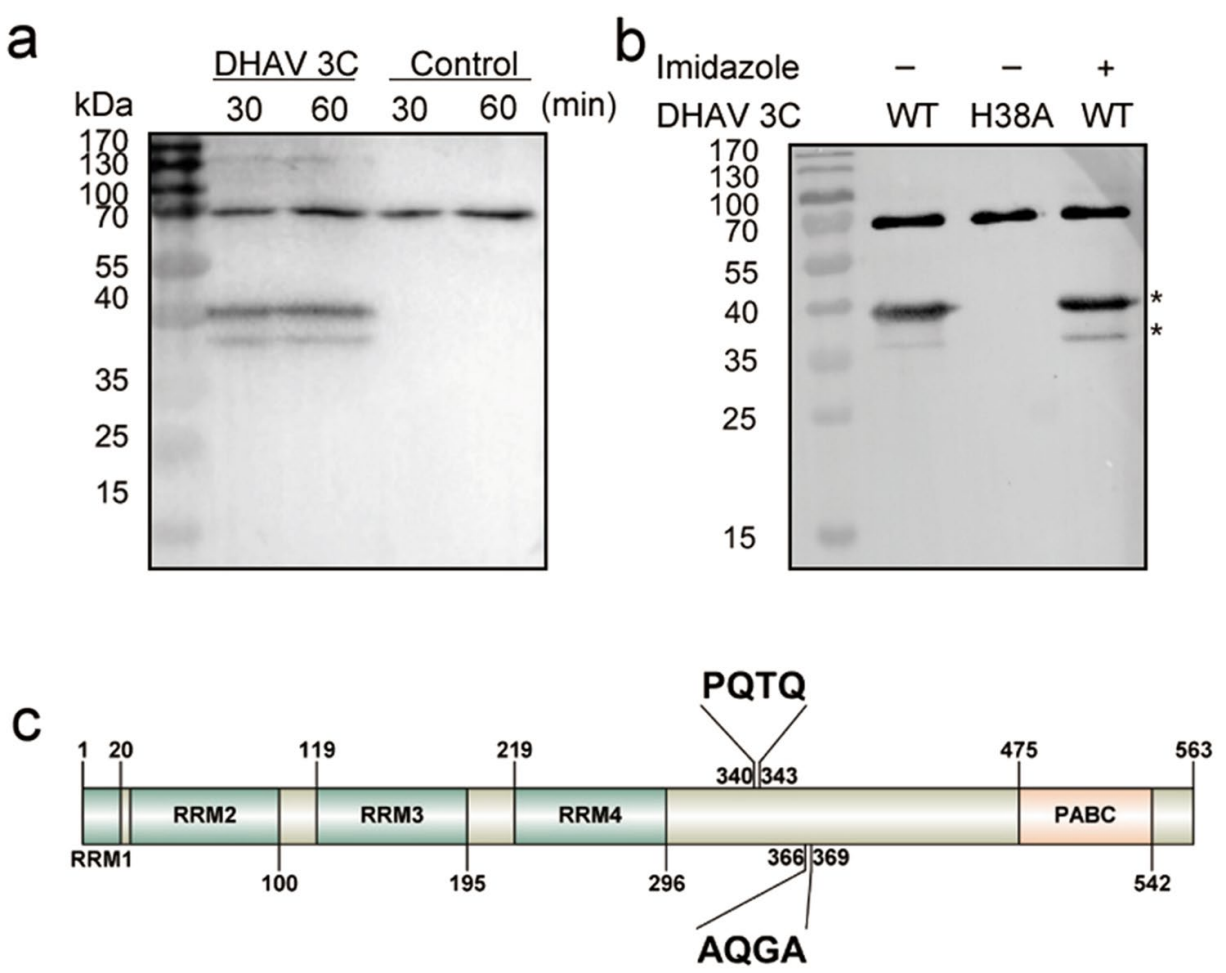
Cleavage of PABP by the DHAV 3C protease. (**a**) Cleavage of PABP by the DHAV 3C protease in uninfected DEF cells. (**b**) Cleavage of recombinant PABP by DHAV 3C protease *in vitro*. (**c**) Schematic diagram of PABP structure showing the known functional domains and predicted localization of the protease cleavage sites.

Picornaviruses, including PV, EMCV, HAV, and FMDV, have been reported to cleave PABP with 3C protease. To evaluate whether PABP is a direct substrate of DHAV 3C protease, the cleavage reaction was performed, and PABP cleavage products were identified by immunoblotting with anti-PABP antibody. The total DEF cellular proteins were harvested with cell lysis buffer and incubated with the recombinant 3C protease at 37 °C for 30 min and 60 min. After incubation with 3C protease, two bands with an apparent molecular mass of 37 kDa reacted with the anti-PABP antiserum (Fig. 4a). In contrast, these two bands were not detected in the control group without 3C protease. The results demonstrated that 3C protease was sufficient to generate PABP cleavage without assistance from other viral proteins. Overall, these findings suggest that PABP is a direct substrate of 3C protease, which is consistent with the cleavage of PABP by other picornaviruses in cells.

### Recombinant PABP is cleaved *in vitro* by DHAV 3C protease

Evidence from previous experiments suggests that endogenous PABP in DEF cells can be cleaved specifically by DHAV 3C protease. To determine whether DHAV 3C protease can cleave PABP without the assistance of any other eukaryotic cellular factors, recombinant PABP was expressed in *E. coli* with an N-terminal hexahistidine tag and purified using Ni^2+^ ion-based affinity chromatography. Recombinant PABP was incubated with 3C protease or mutant 3C (H38A) for 12 h at 4 °C, and the cleavage products were fractioned by 12.5% SDS-PAGE and immunoblotted with anti-PABP monoclonal antibody. The recombinant H38A mutant of 3C was used as a negative control. Two bands of ~36 kDa and ~38 kDa were detected specifically with anti-PABP serum, while only full-length PABP was observed in the control group (Fig. 4b). Furthermore, imidazole in cleavage buffer had no influence on the proteolysis of PABP by 3C protease. The cleavage products of DHAV 3C protease were compared with the cleavage sites of PV 3C protease (Supplementary Table 1), and it appeared that Q341T and Q367G generated cleavage products consistent in molecular size with the observed products (Fig. 4c). This result suggested that DHAV 3C protease could cleave PABP in the absence of any additional viral proteins or cellular proteins.

### Identification of the cleavage sites of DHAV 3C protease on PABP

**Figure 5:**
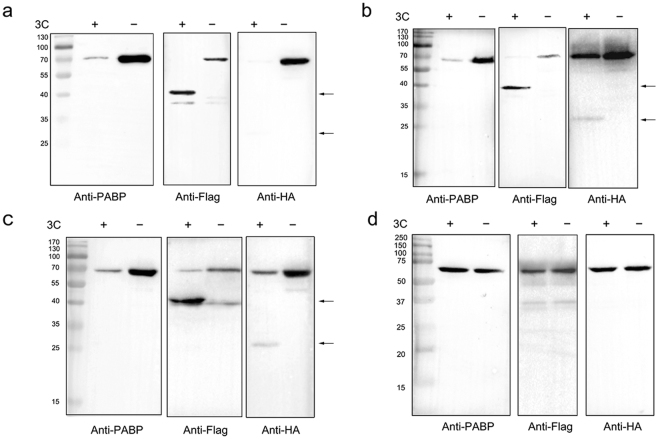
Cleavage of recombinant wild-type (WT) PABP and mutants by 3C protease *in vitro*. (**a**) Recombinant PABP with Flag at N-terminal and HA at C-terminal was cleaved by DHAV 3C protease. (**b**) Recombinant DHAV 3C protease-mediated PABP mutant (G368N) cleavage. (**c**) Recombinant DHAV 3C protease-mediated PABP mutant (Q341A) cleavage. (**d**) Recombinant of DHAV 3C protease mediated PABP mutant (Q367N) cleavage. Reaction products were fractionated by 12.5% SDS-PAGE and analysed by Western blotting with anti-PABP, anti-Flag or anti-HA antibodies. Anti-Flag antibody was used to visualize the N-terminal fragment of PABP, while anti-HA antibody was used to visualize the C-terminal fragment of PABP. The cleaved fragments were indicated with the black arrows.

To confirm the exact cleavage sequence within PABP that is recognized by DHAV 3C protease, the PABP amino acid sequence was analysed to search for a typical cleavage junction. Considering the molecular weight of the cleavage products, the suspected cleavage sites glutamine 341-threonine 342 and glutamine 367-glycine 368 are both located within the C-terminal linker region (Fig. 4c). To further identify the precise cleavage site in duck PABP, single amino acid mutations were generated at Q341A, Q367N and G368N in recombinant PABP with an N-terminal Flag tag and a C-terminal haemagglutinin (HA) tag. *In vitro* cleavage reactions were then performed with bacterially expressed mutant PABP proteins and analysed by Western blotting using anti-PABP antibody to clarify the expression of PABP and using two tag antibodies to detect the cleavage products. The tagged wild-type (WT) PABP was cleaved by DHAV 3C protease (Fig. 5a). The G368N mutant of PABP did not abolish proteolysis by 3C protease, as the accumulation of cleavage products of ~38 kDa and ~25 kDa was detected (Fig. 5b). The molecular weights of these cleavage fragments were similar to those of the cleavage products of WT PABP (Fig. 5a). Incubation of the expressed Q341A mutant of PABP with recombinant 3C protease yielded fragments with apparent molecular weights of ~25 kDa and ~38 kDa (Fig. 5c), while PABP Q367N was resistant to proteolytic cleavage (Fig. 5d). These results revealed that Q367/G368 was the PABP cleavage site. These recombinant PABP mutants were all recognized by mouse monoclonal antibodies directed against PABP. The experiments were repeated three times with similar results. Cleavage at AQ↓GA in duck PABP is consistent with the cleavage specificity of picornaviral 3C proteases, for which the principal determinants for cleavage are Gln-Gly (Q-G) amino acid pairs. The result is also consistent with the cleavage specificity of PV 3C protease and EMCV 3C protease, whose sites are Q437/G438. The sequence of the flexible proline-rich linker within PABP is conserved among different genera (Supplementary Figure S1).

### The role of PABP in DHAV replication

**Figure 6:**
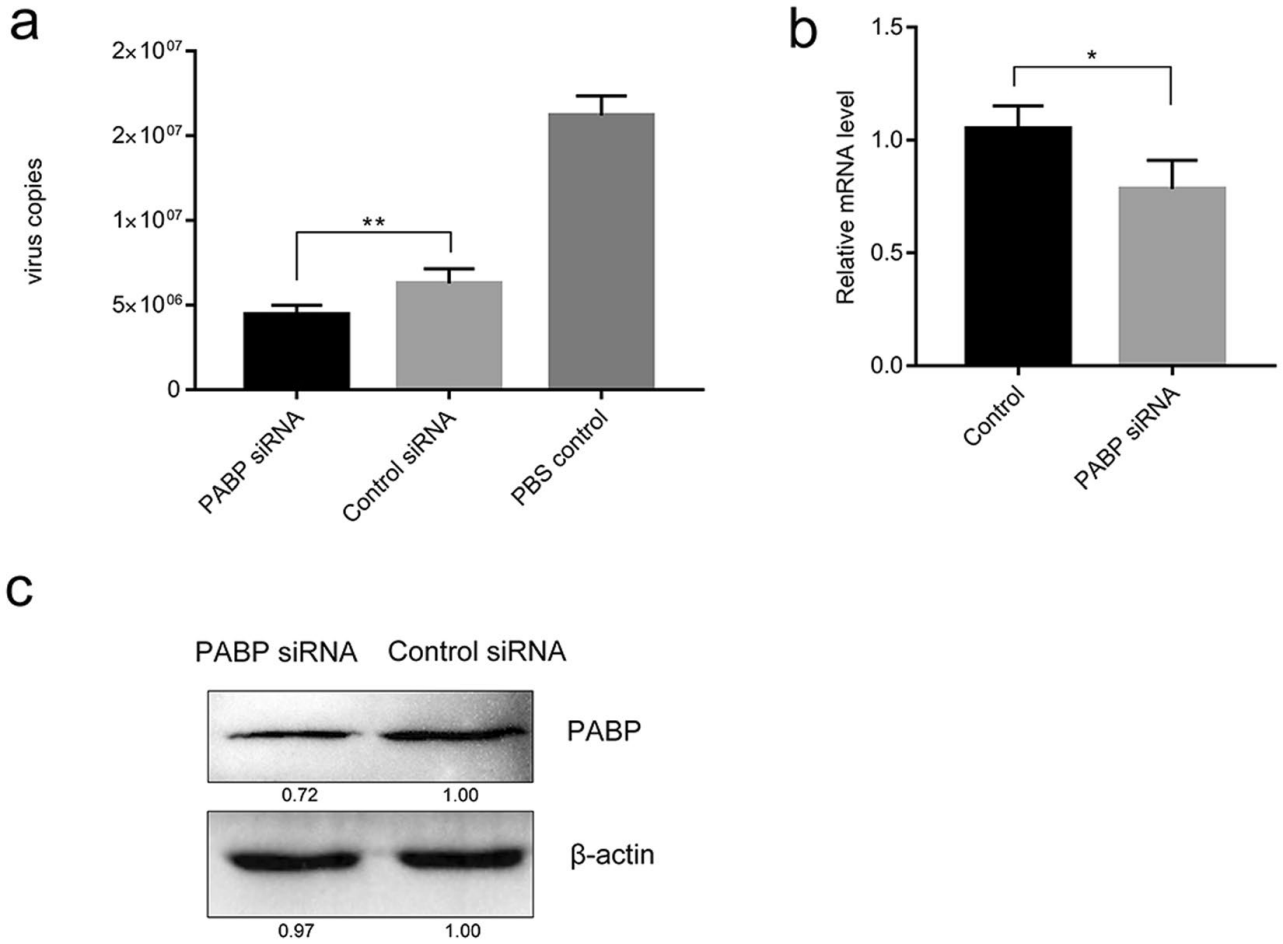
Amount of viral RNA in siRNA-mediated PABP knockdown cells. (**a**) At 12 hpi, the copy numbers of VP0 RNA were detected with a one-step rRT-PCR assay and compared with those of the control siRNA and the PBS control groups. (**b**) Relative PABP mRNA levels in the cells transfected with PABP siRNA or control siRNA and infected with DHAV were measured by performing the 2^−ΔΔCt^ method with RT-PCR. *P < 0.05; **P < 0.01. (**c**) The PABP protein level in the same batch of cells was determined by immunoblotting. Cell lysates were detected with anti-PABP and anti-β-actin antibodies.

**Figure 7:**
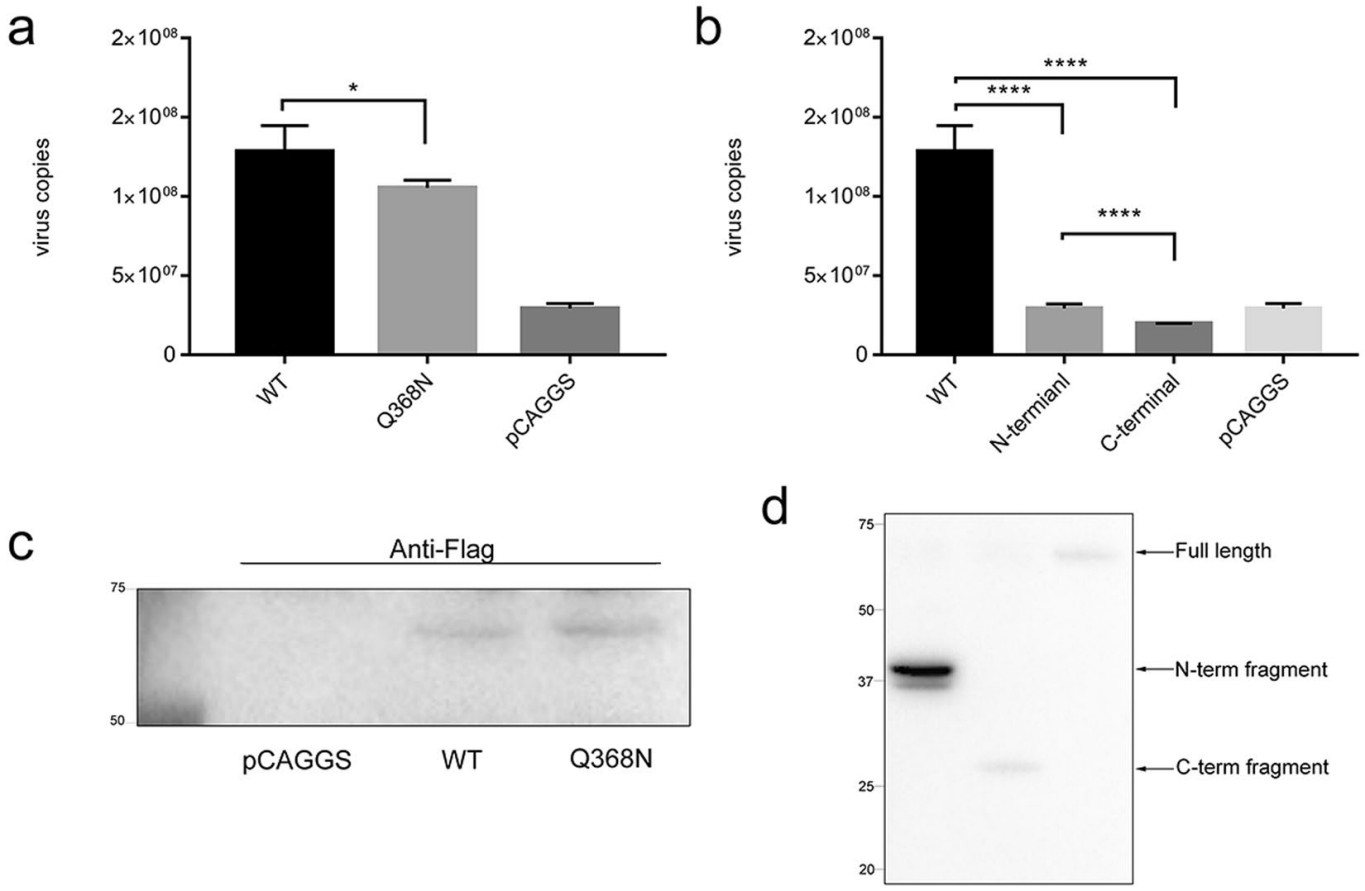
Amount of viral RNA in DEF cells overexpressing WT PABP, the Q367N cleavage-resistant variant and truncated PABP. (**a**) DEF cells overexpressing WT PABP or the Q367N cleavage-resistant variant were infected with DHAV. At 12 hpi, total RNA was isolated and the copy numbers of VP0 RNA were detected with a one-step rRT-PCR assay and compared with those cells transfected with the empty vector. (**b**) DEF cells expressing the N-terminal domain (NTD) of PABP and C-terminal domain (CTD) of PABP were infected with DHAV. At 12 hpi, total RNA was isolated and the copy numbers of VP0 RNA were detected and compared with those cells transfected empty vector. *P < 0.05; ****P < 0.0001. (**c**) The cell lysates in the same batch of cells expressing WT PABP and the Q367N cleavage-resistant variant were analysed by immunoblotting. Cell lysates were detected with anti-Flag antibody. (**d**) The lysates of cells expressing NTD of PABP and CTD of PABP were analysed by immunoblotting. Cell lysates were detected with anti-Flag antibody.

We attempted to determine whether PABP played a direct role in DHAV RNA replication. DEF cells were transfected with PABP siRNA and control siRNA using Lipofectamine 2000. Then, the transfected cells were infected with DHAV (TCID_50_ of 1000), and cells were harvested at 12 hpi. The viral RNA was measured by real-time fluorescence quantitative reverse transcription-polymerase chain reaction (rRT-PCR). The virus load was reduced in DHAV-infected cells treated with PABP siRNA compared to the virus level in the control cells at 12 hpi (Fig. 6a). To evaluate the knockdown efficiency of siRNA, we extracted RNAs from cells treated with PABP siRNA for same time and performed real-time RT-PCR to measure the endogenous mRNA level of PABP. Compared to transfection with control siRNA, we observed a nearly 25% reduction in the PABP abundance after transfection with PABP siRNA (Fig. 6b). Similar results were obtained from an immunoblot experiment from the same batch of cells (Fig. 6c). The results suggested that PABP knockdown decreased the RNA replication of DHAV; therefore, we hypothesized that PABP was required for the DHAV infection in DEF cells. DEF cells transfected with plasmids overexpressing PABP, plasmids expressing the Q368N cleavage-resistant mutant, or the pCAGGS vector were infected with DHAV. In contrast to cells expressing WT PABP, a slight reduction in the amount of viral RNA was found in cells expressing the PABP Q368N mutant at 12 hpi (Fig. 7a). To identify the function of the cleavage fragments, we expressed truncated PABP (N-terminal domain and C-terminal domain) in PABP knockdown cells respectively before infection with DHAV. The data indicated that the C-terminal domain (CTD) had a negative effect on viral replication in contrast to the N-terminal domain (NTD) of PABP (Fig. 7b). We harvested transfected cells for immunoblotting to detect the expression of PABP and cleavage fragments (Fig. 7c and d).

## Discussion

Due to their limited genomes, many viruses depend on cellular translation factors to meet their requirements for reproduction. Picornaviruses adopt various strategies to hijack cellular resources. The significant role of picornaviral 3C protease in guaranteeing the viral replication cycle has been previously reviewed^35^. In this report, it was found that the multifunctional RNA-binding protein PABP is a substrate for DHAV 3C protease. Full-length PABP was observed to decrease at 3 hpi and increase again as soon as 6 hpi. Moreover, DHAV 3C protease was essential and sufficient for PABP cleavage without other viral proteins or cellular factors. Recombinant PABP and single amino acid substitution were utilized to identify the cleavage site Q367/G368 within the C-terminal linker. PABP was also found to be cleaved by other picornavirus enzymes, such as PV proteases and the HAV and EMCV 3C proteases. In addition, PABP is a target for calicivirus and for retroviruses whose proteases mediate proteolytic cleavage^36,37^. The results of our experiments clearly indicated that the interaction between PABP and 3C protease played a potential role in DHAV infection.

Most picornaviruses initiate the translation of viral RNA by the cleavage of the translation initiation factor eIF4G or of PABP. For instance, eIF4G can be cleaved by PV 2A protease^38^, and PABP can be degraded by the 2A protease and 3C protease of PV and by the 2A protease of coxsackievirus B3^8,28,29,39^. During PV infection, the viral 3C protease cleaves PABP at three sites (Q537/G538, Q437/G438 and Q413/T414), and the viral 2A protease also cleaves PABP^39^. PABP is believed to stimulate picornavirus translation via interaction with eIF4G^40^. In addition to the PABP-eIF4GI interaction, PABP binds to eIF4B, and eIF4B can bind the PV IRES, directly participating in translation initiation^8,41^. Notably, no cleavage of eIF4G was detected in cells infected with EMCV, while the 3C protease of EMCV was sufficient to stimulate PABP cleavage at WTAQ437/G438 during infection^42^. Similarly, eIF4G was not a substrate of the 3C protease of HAV, but PABP was cleaved by the HAV 3C protease at or near Q415/T416, which is relevant to RNA template switching^9^. Interestingly, intact eIF4G is required for the IRES-mediated translation of DHAV with the expression of SVDV 2A protease^12^. Remarkably, there are multiple substrates for 2A protease in baby hamster kidney (BHK) cells. Here, DHAV 3C protease was demonstrated to cleave PABP at WTAQ367/G368. PABP consists of four RNA-recognition motifs (RRM1-4), a flexible proline-rich linker and a CTD. The 3C proteases of HAV, EMCV and DHAV could recognize cleavage sites in PABP that were mapped within the linker separating the protein-interacting domain from the RNA-binding domains. Based on the results shown below, we speculate that DHAV, HAV and EMCV might share similar approaches to recognizing PABP, whereas PV has a distinctive approach.

Furthermore, some members of the *Picornavirus* family induce shutoff of the host cell efficiently by suppressing cellular protein synthesis with the cooperation of viral L protease or 2A protease. For instance, 2A protease from diverse enteroviruses and L protease from aphthoviruses cleave eIF4G^29,43–45^. Unlike the enteroviruses and aphthoviruses, DHAV 5′UTR-directed translation is insensitive to eIF4G cleavage^23^. The 3C protease in DHAV is the only protease for processing the polyprotein, and the EMCV 3C protease is likewise the only protease of EMCV. Therefore, the 3C protease of DHAV exhibits various strategies that inhibit the translation of host cell in favour of viral replication. EMCV (type II IRES), HAV (type III IRES) and DHAV (type IV IRES)^46^ have different types of IRESs. In this study, DHAV infection was shown to stimulate the PABP decrease before 6 hpi, but no PABP fragments were detected. As noted previously, the monoclonal anti-PABP antibody could not recognize any cleavage products of PABP generated by enterovirus proteases. Nonetheless, the PABP antibody could detect the cleavage product in the cases of uninfected cells incubated with 3C protease and of recombinant PABP incubated with 3C protease *in vitro* in the absence of other viral proteins or eukaryotic cellular cofactors. These results suggest that PABP cleavage products are generated by the 3C protease of DHAV *in vitro*. Two truncated fragments were detected, as the anti-Flag serum could interact with the N-terminal cleavage product of PABP and the anti-HA serum could recognize the C-terminal cleavage product. The N-terminal fragment of PABP generated by HAV 3C protease was proven to inhibit IRES-dependent translation and enhance the binding capacity to the RNA structural element pY1, which is essential for viral replication^9^. PABP cleavage was identified as a regulatory factor in switching the RNA template from viral translation to RNA synthesis. Similarly, PABP cleavage was required for efficient EMCV replication^14^. To better understand the exact molecular mechanisms, further studies are necessary, including identification of the role of the PABP N-terminal fragment in IRES-dependent translation.

PABP binding to the 3′-poly(A) tail stimulates cellular translation initiation. In contrast, viruses take advantage of RNA-RNA interactions with IRES and RNA-protein interactions requiring cellular factors for translation^47^. CVB3 can circularize through a 5′UTR-protein-protein-3′UTR mechanism^48^. The poly(A) tail contributes significantly to the translation of PV, EMCV and HAV^49^. In addition, the regulation of HCV RNA translation is closely related to poly(U/UC) in the 3′UTR^50^. DHAV contains the longest 3′UTR in the *Picornavirus* family. However, the deletion of the 3′UTR had no effect on DHAV IRES-mediated translation initiation with a bicistronic reporter minigenome system^20^.

Although our findings suggested that PABP was targeted by DHAV infection, perhaps an intriguing question that remains unclear is how DHAV benefits from the cleavage of PABP. What mediates the switch from translation to viral replication in DHAV? It will be significant to investigate how the PABP cleavage contributes to the steps of the viral life cycle. Researching how DHAV manipulates the cleavage of PABP will lay the groundwork for understanding various translation strategies of viruses and for the development of antiviral medicines.

## Methods

### Cells and virus

DEF cells were grown in a spinner culture in minimum essential medium (MEM) supplemented with 10% newborn calf serum and penicillin-streptomycin (Gibco). To prepare the virus stocks, DHAV was grown in DEF cells for 72 h. Precipitates were sedimented by centrifugation at 4000 rpm for 10 min. The virus suspension was frozen at −70 °C. The virus titre was measured by 50% tissue culture infective doses [TCID_50_] and determined by the incubation of serial 10-fold dilutions of the virus with DEF cells in 96-well microtiter plates. After 60 min at 37 °C in a humidified 5% CO_2_ incubator, the inocula were removed, and the cells were washed with phosphate-buffered saline (PBS). The DEF cells were cultured in minimum essential medium supplemented with 2% serum. Wells with uninfected cells were included in each plate as negative controls. TCID_50_ was determined at 48 h.

### Plasmids

The synthetic oligonucleotide primers DHAV-3C-*Nde* I (+) and DHAV-3C-*Hind* III (−) were used to generate PCR fragments containing DHAV amino acids 4846 to 5388, flanked by *Nde* I upstream and *Hind* III downstream. The restriction sites were then used to clone the DHAV 3C gene into the pET32a vector through ligation. Plasmids pET32a-3Cwt and pET32a-3Cm-H38A expressed, respectively, the DHAV catalytic 3C protease and the catalytically inactive 3C mutant containing an amino acid substitution in the catalytic triad within the active site (unpublished materials).

According to the manufacturer’s protocols, the RNAiso Plus reagent (TaKaRa) was used to isolate duck cellular RNA. The RNA isolated from DHAV was used for reverse transcription with the PrimeScript^TM^ RT Reagent Kit (TaKaRa) to produce cDNA as a template. Duck PABP was PCR amplified from cDNA with the primers given in Table 1. The plasmid pET28-PABP expressed a His-tag at the N-terminal of PABP. To construct N-terminally Flag-tagged and C-terminally HA-tagged PABP, the amplification products were digested with *Nde* I and *Bgl* II and then inserted into the pET32a vector to generate pET32a-Flag-PABP-HA. The variants of PABP were constructed using overlapping PCR with the specified primers. To generate pCAGGS-Flag-PABP-HA, pCAGGS-Flag-PABP-NTD-HA and pCAGGS-Flag-PABP-CTD-HA, the PABP cDNA was amplified with the specified primers and integrated into the pCAGGS vector with the One Step Cloning Kit (Vazyme).

**Table 1:** Primers used in this study.

Primer	Sequence (5′-3′)
pCAGGS-PABP-F	CATCATTTTGGCAAAGAATTCGCCACCATG*GATTACAAGGATGACGACGATAAG*ATGAACTTTGATGTCATTAAAGGC
pCAGGS-PABP-R	TTGGCAGAGGGAAAAAGATCTTTA*AGCGTAGTCTGGGACGTCGTATGGGTA*GGGGTTATTAACTGCCTTTT
pCAGGS-PABP-Q367-R	TTGGCAGAGGGAAAAAGATCTTTA*AGCGTAGTCTGGGACGTCGTATGGGTA*CTGAGCAGTCCAGCGAGGAC
pCAGGS-PABP-G368-F	CATCATTTTGGCAAAGAATTCGCCACCATG*GATTACAAGGATGACGACGATAAG*GGTGCCAGACCTCATCCATT
pET28-PABP-F	CATATGATGAACTTTGATGTCATTAAAGGC
pET28-PABP-R	AAGCTTTTATGGGGTTATTAACTGCCTTTT
pET32a-Flag-PABP-HA-F	GGAATTCCATATG *GATTACAAGGATGACGACGATAAG*ATGAACTTTGATGTCATTAAAG
pET32a-Flag-PABP-HA-R	TTGGCAGAGGGAAAAAGATCTTTA*AGCGTAGTCTGGGACGTCGTATGGGTA*GGGGTTATTAACTGCCTTTT
G368N-F	TCGCTGGACTGCTCAG**AAT**GCCAGACC
G368N-R	GGTCTGGC**ATT**CTGAGCAGTCCAGCGA
Q341A-F	ATCCCA**GCG**ACTCAGAACCGTGCT
Q341A-R	CGGTTCTGAGT**CGC**TGGGATAGCT
Q367N-F	TCGCTGGACTGCT**AAT**GGTGCCAGACC
Q367N-R	GGTCTGGCACC**ATT**AGCAGTCCAGCGA
β-actin-RTPCR-F	TACGCCAACACGGTGCTG
β-actin-RTPCR-R	GATTCATCATACTCCTGCTTG
PABP-RTPCR-F	AAGGCTTCGGCTTCGTTAGTT
PABP-RTPCR-R	GATCCTGTCCTGCTTCATTTGC

### Reagents and antibodies

RNAiso plus, the PrimeScript^TM^ RT Reagent Kit (Perfect Time) and SYBR®Premix Ex Taq™ II were purchased from TAKARA. PABP siRNA (sc-36169) and control siRNA (sc-37007) were purchased from Santa Cruz. Mouse monoclonal anti-PABP [10E10] (ab6125) and β-actin were purchased from Abcam, and antibodies against Flag and HA were purchased from Beyotime and TransGen Biotech, respectively. Lipofectamine 2000 was purchased from Thermo Fisher as a transfection reagent.

### Expression of recombinant protein

The cDNA encoding duck PABP (aa 1 to 557) was amplified using reverse transcription-PCR (RT-PCR) with the duck RNA (GenBank accession no. XM_013106433.1) as the template. Next, the amplification products were digested with the *Nde* I and *Hind* III restriction enzymes and cloned into the pET28a expression vector to produce PABP with a His * 6 tag at the N-terminus. The coding region was verified by DNA sequencing, and the construct was transformed into *E. coli* BL21 cells for expression. The transformed cells were cultured at 37 °C in LB medium containing 100 mg/l ampicillin. After the optical density at 600 nm (OD600) of the culture reached 0.6, protein expression was induced with 0.5 mM isopropyl-β-D-thiogalactoside (IPTG) for 16 h. The cells were harvested by centrifugation, resuspended in lysis buffer (50 mM Na_2_H_2_PO_4_.2H_2_O [pH 8.0], 300 mM NaCl) and ultrasonically decomposed on ice. The lysate was clarified by centrifugation at 12,000 rpm for 10 min at 4 °C. The supernatant was loaded onto a 5 ml Ni-nitrilotriacetic acid (NTA) column equilibrated in lysis buffer. Nonspecific contaminants were removed by washing twice with buffer containing 10 mM and then 20 mM imidazole. The PABP protein was subsequently eluted in lysis buffer with 200 mM imidazole. The purified PABP protein was concentrated to approximately 1 to 2 mg/ml in buffer containing 20 mM Tris-HCl (pH 7.0), 150 mM NaCl, 1 mM EDTA, 5 mM DTT, and 10% glycerol.

### *In vitro* cleavage reactions

For the cleavage of PABP in cell fractions, 20 μl of extract was incubated with DHAV 3C protease (1 mg/ml). The cleavage products were detected by immunoblot analysis using anti-PABP. For recombinant PABP, 200 ng of purified recombinant PABP and DHAV 3C protease (final concentration, 1 mg/ml) or lysate prepared from bacteria expressing the plasmid pET32a were incubated at 16 °C for 12 h in cleavage buffer (20 mM Tris-HCl (pH 7.0), 150 mM NaCl, 1 mM EDTA, 5 mM DTT, 10% glycerol)^51^. The reaction was stopped by the addition of 5 * SDS-PAGE sample buffer, followed by boiling. The reaction products were fractionated by SDS-PAGE and analysed by Western blotting with anti-PABP, anti-Flag and anti-HA antibodies.

### Indirect IF microscopy

DEF cell monolayers were grown on glass coverslips to 50–70% confluence and then infected with DHAV (1000 TCID_50_). At various time points post-infection, the cells were rinsed (three times) with cold PBS and then fixed in 4% paraformaldehyde for 30 min. The DEF cells were permeabilized (0.2% Triton X-100 for 15 min), incubated with blocking solution (5% bovine serum albumin (BSA) in PBS with Tween 20 (PBST) for 60 min), and then sequentially incubated with anti-PABP antibody and anti-3C antibody as primary antibodies and FITC-conjugated goat anti-mouse IgG and Texas-conjugated goat anti-rabbit IgG as secondary antibodies, respectively, for 1 h. The cells were then washed twice in PBST and treated with 4′,6-diamidino-2-phenylindole (DAPI) (Beyotime). Images were captured using an 80i upright microscope (Nikon) and a SPOT Flex camera.

### siRNA-mediated knockdown of PABP

Pre-duplexed small interfering RNAs (siRNAs) were obtained from Santa Cruz. DEF cells were seeded to 70–80% confluency in 12-well tissue culture plates. For a 12-well tissue culture plate, 10 µM of control or PABP-specific siRNA were transfected using Lipofectamine 2000 (Invitrogen). The transfection complexes were incubated with the cells for 6 h. Twenty-four hours after transfection, DEF cells were infected with DHAV (1000 TCID_50_) for 12 h. After removal of the MEM, the DEF cells were washed using PBS three times and harvested. The number of viral copies was detected by methods previously established in our laboratory^52^. Same batches of the cells were collected to determine the efficiency of PABP knockdown by RT-PCR and Western blot analysis. The mRNA level of PABP and a housekeeping gene (β-actin) were measured using RT-PCR with specific primers (Table 1).

## Electronic supplementary material

Supplementary Information

## References

[1] Sonenberg N, Hinnebusch AG (2009). Regulation of translation initiation in eukaryotes: mechanisms and biological targets. Cell.

[2] Mangus DA, Evans MC, Jacobson A (2003). Poly(A)-binding proteins: multifunctional scaffolds for the post-transcriptional control of gene expression. Genome Biol..

[3] Gorgoni B, Gray NK (2004). The roles of cytoplasmic poly(A)-binding proteins in regulating gene expression: a developmental perspective. Brief. Funct. Genomic Proteomic..

[4] Bushell M (2001). Disruption of the interaction of mammalian protein synthesis eukaryotic initiation factor 4B with the poly(A)-binding protein by caspase-and viral protease-mediated cleavages. J. Biol. Chem..

[5] Wang Z, Day N, Trifillis P, Kiledjian M (1999). An mRNA stability complex functions with poly(A)-binding protein to stabilize mRNA *in vitro*. Mol. Cell Biol..

[6] Lee KM, Chen CJ, Shih SR (2017). Regulation mechanisms of viral IRES-driven translation. Trends Microbiol..

[7] Spector DH, Villa-Komaroff L, Baltimore D (1975). Studies on the function of polyadenylic acid on poliovirus RNA. Cell.

[8] Bonderoff JM, Larey JL, Lloyd RE (2008). Cleavage of poly(A)-binding protein by poliovirus 3C proteinase inhibits viral internal ribosome entry site-mediated translation. J. Virol..

[9] Zhang B, Morace G, Gauss-Muller V, Kusov Y (2007). Poly(A) binding protein, C-terminally truncated by the hepatitis A virus proteinase 3C, inhibits viral translation. Nucleic Acids Res..

[10] Belsham GJ, McInerney GM, Ross-Smith N (2000). Foot-and-mouth disease virus 3C protease induces cleavage of translation initiation factors eIF4A and eIF4G within infected cells. J. Virol..

[11] de Breyne S, Bonderoff JM, Chumakov KM, Lloyd RE, Hellen CU (2008). Cleavage of eukaryotic initiation factor eIF5B by enterovirus 3C proteases. Virology.

[12] Pan M (2012). Duck hepatitis A virus possesses a distinct type IV internal ribosome entry site element of picornavirus. J. Virol..

[13] Kuyumcu-Martinez NM, Van Eden ME, Younan P, Lloyd RE (2004). Cleavage of poly(A)-binding protein by poliovirus 3C protease inhibits host cell translation: a novel mechanism for host translation shutoff. Mol. Cell Biol..

[14] Kobayashi M, Arias C, Garabedian A, Palmenberg AC, Mohr I (2012). Site-specific cleavage of the host poly(A) binding protein by the encephalomyocarditis virus 3C proteinase stimulates viral replication. J. Virol..

[15] Wen XJ (2014). Detection, differentiation, and VP1 sequencing of duck hepatitis A virus type 1 and type 3 by a 1-step duplex reverse-transcription PCR assay. Poult. Sci..

[16] Ou, X. *et al*. The neglected avian hepatotropic virus induces acute and chronic hepatitis in ducks: an alternative model for hepatology. *Oncotarget* (2017). 10.18632/oncotarget.19003 PMC5669852 29137226

[17] Shen Y (2015). Development of an indirect ELISA method based on the VP3 protein of duck hepatitis A virus type 1 (DHAV-1) for dual detection of DHAV-1 and DHAV-3 antibodies. J. Virol. Methods.

[18] Wen X. *et al*. Molecular epidemiology of duck hepatitis a virus types 1 and 3 in China, 2010–2015. *Transbound Emerg Di*s. (2017). 10.1111/tbed.12741 29076646

[19] Pattison, M. *Poultry diseases*. (Elsevier Health Sciences, 2008).

[20] Liang R (2015). Duck hepatitis A virus serotype 1 minigenome: a model for studying the viral 3′UTR effect on viral translation. Virus Genes.

[21] Cao J (2016). The 2A2 protein of Duck hepatitis A virus type 1 induces apoptosis in primary cell culture. Virus Genes..

[22] Kim MC (2007). Recent Korean isolates of duck hepatitis virus reveal the presence of a new geno- and serotype when compared to duck hepatitis virus type 1 type strains. Arch. Virol..

[23] Yang X (2017). Structures and Corresponding Functions of Five Types of Picornaviral 2A Proteins. Front Microbiol..

[24] Liu G, Yanguez E, Chen Z, Li C (2011). The duck hepatitis virus 5′-UTR possesses HCV-like IRES activity that is independent of eIF4F complex and modulated by downstream coding sequences. Virol. J..

[25] Pulido MR, Serrano P, Saiz M, Martinez-Salas E (2007). Foot-and-mouth disease virus infection induces proteolytic cleavage of PTB, eIF3a,b, and PABP RNA-binding proteins. Virology.

[26] Gradi A (2004). Cleavage of eukaryotic translation initiation factor 4GII within foot-and-mouth disease virus-infected cells: identification of the L-protease cleavage site *in vitro*. J. Virol..

[27] Foeger N, Kuehnel E, Cencic R, Skern T (2005). The binding of foot-and-mouth disease virus leader proteinase to eIF4GI involves conserved ionic interactions. FEBS J..

[28] Joachims M, Van Breugel PC, Lloyd RE (1999). Cleavage of poly(A)-binding protein by enterovirus proteases concurrent with inhibition of translation *in vitro*. J. Virol..

[29] Kerekatte V (1999). Cleavage of Poly(A)-binding protein by coxsackievirus 2A protease *in vitro* and *in vivo*: another mechanism for host protein synthesis shutoff?. J. Virol..

[30] Krausslich HG, Nicklin MJ, Toyoda H, Etchison D, Wimmer E (1987). Poliovirus proteinase 2A induces cleavage of eucaryotic initiation factor 4F polypeptide p220. J. Virol..

[31] Monette A (2013). Dual mechanisms of translation initiation of the full-length HIV-1 mRNA contribute to gag synthesis. PLoS One.

[32] Silvera D, Formenti SC, Schneider RJ (2010). Translational control in cancer. Nat. Rev. Cancer.

[33] Gorlach M, Burd CG, Dreyfuss G (1994). The mRNA poly(A)-binding protein: localization, abundance, and RNA-binding specificity. Exp. Cell Res..

[34] Sharma R, Raychaudhuri S, Dasgupta A (2004). Nuclear entry of poliovirus protease-polymerase precursor 3CD: implications for host cell transcription shut-off. Virology.

[35] Sun D, Chen S, Cheng A, Wang M (2016). Roles of the picornaviral 3C proteinase in the viral life cycle and host cells. Viruses.

[36] Kuyumcu-Martinez M (2004). Calicivirus 3C-like proteinase inhibits cellular translation by cleavage of poly(A)-binding protein. J. Virol..

[37] Alvarez E, Castello A, Menendez-Arias L, Carrasco L (2006). HIV protease cleaves poly(A)-binding protein. Biochem. J..

[38] Zamora M, Marissen WE, Lloyd RE (2002). Multiple eIF4GI-specific protease activities present in uninfected and poliovirus-infected cells. J. Virol..

[39] Kuyumcu-Martinez NM, Joachims M, Lloyd RE (2002). Efficient cleavage of ribosome-associated poly(A)-binding protein by enterovirus 3C protease. J. Virol..

[40] Svitkin YV (2001). Poly(A)-binding protein interaction with elF4G stimulates picornavirus IRES-dependent translation. RNA.

[41] Ochs K (2002). Interaction of translation initiation factor eIF4B with the poliovirus internal ribosome entry site. J. Virol..

[42] Mosenkis J (1985). Shutoff of host translation by encephalomyocarditis virus infection does not involve cleavage of the eucaryotic initiation factor 4F polypeptide that accompanies poliovirus infection. J. Virol..

[43] Chau DH (2007). Coxsackievirus B3 proteases 2A and 3C induce apoptotic cell death through mitochondrial injury and cleavage of eIF4GI but not DAP5/p97/NAT1. Apoptosis.

[44] Medina M, Domingo E, Brangwyn JK, Belsham GJ (1993). The two species of the foot-and-mouth disease virus leader protein, expressed individually, exhibit the same activities. Virology.

[45] Devaney MA, Vakharia VN, Lloyd RE, Ehrenfeld E, Grubman MJ (1988). Leader protein of foot-and-mouth disease virus is required for cleavage of the p220 component of the cap-binding protein complex. J. Virol..

[46] Asnani M, Kumar P, Hellen CU (2015). Widespread distribution and structural diversity of Type IV IRESs in members of *Picornaviridae*. Virology.

[47] Bai Y, Zhou K, Doudna JA (2013). Hepatitis C virus 3′UTR regulates viral translation through direct interactions with the host translation machinery. Nucleic Acids Res..

[48] Souii A, M’hadheb-Gharbi MB, Gharbi J (2015). Cellular proteins act as bridge between 5′ and 3′ ends of the Coxsackievirus B3 mediating genome circularization during RNA translation. Curr. Microbiol..

[49] Bergamini G, Preiss T, Hentze MW (2000). Picornavirus IRESes and the poly(A) tail jointly promote cap-independent translation in a mammalian cell-free system. RNA.

[50] Hoffman B, Li Z, Liu Q (2015). Downregulation of viral RNA translation by hepatitis C virus non-structural protein NS5A requires the poly(U/UC) sequence in the 3′ UTR. J. Gen. Virol..

[51] Shang L (2015). Biochemical characterization of recombinant *Enterovirus* 71 3C protease with fluorogenic model peptide substrates and development of a biochemical assay. Antimicrob. Agents Chemother..

[52] Hu Q (2016). A one-step duplex rRT-PCR assay for the simultaneous detection of duck hepatitis A virus genotypes 1 and 3. J. Virol. Methods.

