# Supplementary material for: Cleavage of poly(A)-binding protein by duck hepatitis A virus 3C protease
Source: Sci Rep. 2017 Nov 24;7:16261. doi: 10.1038/s41598-017-16484-1 (PMC5701138; doi:10.1038/s41598-017-16484-1)
Supplement: Supplementary file 1 — Supplementary Information [file 41598_2017_16484_MOESM1_ESM.pdf]

Cleavage of poly (A)-binding protein by duck hepatitis A virus 3C protease

Di Sun[1,2,3+], Mingshu Wang[1,2,3+], Xingjian Wen[1,2,3+], Anchun Cheng[1,2,3*], Renyong Jia[1,2,3], Kunfeng Sun[1,2,3], Qiao Yang[1,2,3], Ying Wu[1,2,3], Dekang Zhu[2,3], Shun Chen[1,2,3], Mafeng Liu[1,2,3], Xinxin Zhao[1,2,3], Xiaoyue Chen[2,3]

[1] Institute of Preventive Veterinary Medicine, Sichuan Agricultural University, Wenjiang, Chengdu, Sichuan, 611130, P.R. China

[2] Key Laboratory of Animal Disease and Human Health of Sichuan Province, Sichuan Agricultural University, Wenjiang, Chengdu, Sichuan, 611130, P.R. China

[3] Avian Disease Research Center, College of Veterinary Medicine, Sichuan Agricultural University, Wenjiang, Chengdu, Sichuan, 611130, P.R. China

[+]These authors contributed equally to this work and should both be considered first authors.

***Corresponding authors:**

Tel: +86 28 86291905

Fax: +86 28 86291905

E-mail address: chenganchun@vip.163.com (A. Cheng)

Supplementary Table

PV $3C^{pro}$ Cleavage Site	DHAV $3C^{pro}$ Cleavage Site	Molecular weight	N-terminal	C-terminal
$AIPQ_{413}/T_{414}$	$AIPQ_{341}/T_{342}$	64.3 kDa	39.7 kDa	24.6 kDa
$WTAQ_{437}/G_{438}$	$WTAQ_{367}/G_{368}$	64.3 kDa	42.7 kDa	21.6 kDa
$VHVQ_{537}/G_{538}$	$VHVQ_{467}/G_{468}$	64.3 kDa	53.3 kDa	11.0 kDa

Supplementary Table 1. Identification of PABP cleavage sites.

Locations of PABP cleavage sites were recognized by poliovirus. These sites were compared to duck hepatitis virus for searching cleavage products.

Supplementary Figures

Figure S1

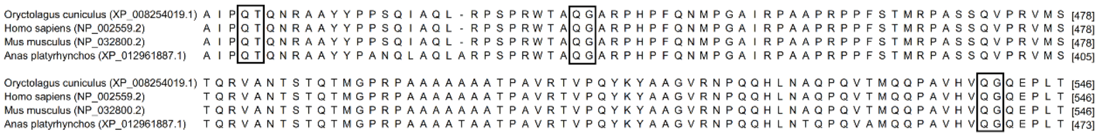

Figure S1. Alignment of the rabbit, mouse, human and duck PABP protein sequences. Numbers to the right of sequences denote the amino acid positions. The frames indicate the cleavage sites of PV 3C protease.

(a) PABP 70kDa

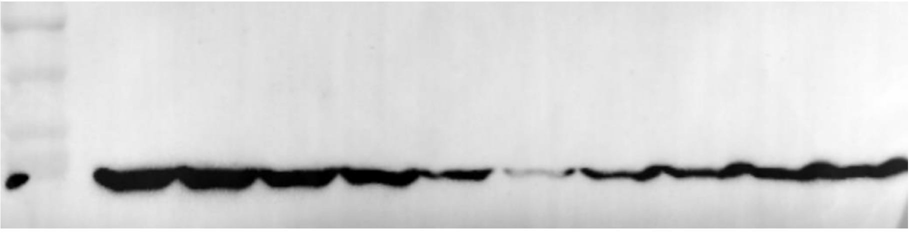

β-actin 42kDa

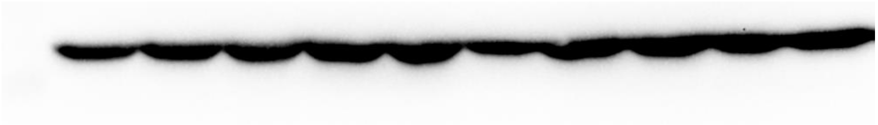

Figure 1. The change in cellular PABP during the DHAV infection of DEF cells. (a) Western blotting analysis was performed to detect the protein expression levels of PABP, with β-actin as the loading control.

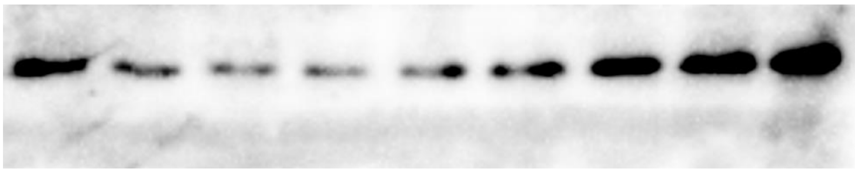

(a) 3C 20kDa

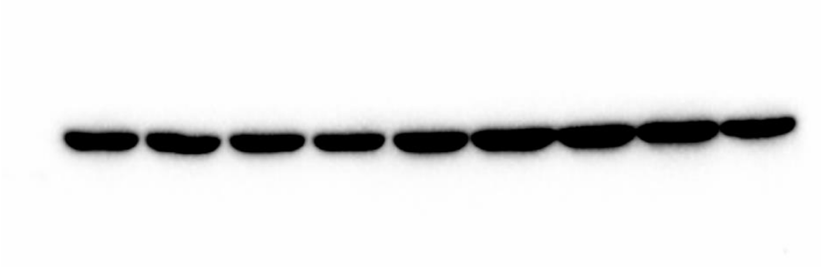

β-actin 42kDa

Figure 2. Accumulation of viral 3C protease in DHAV-infected cells. (**a**) Time course study of DHAV 3C protease expression via immunoblotting. In addition, β-actin was used as a control.

(a) Anti-PABP 75kDa 37kDa 36kDa

DHAV 3C + + - -

M 30 60 30 60 (min)

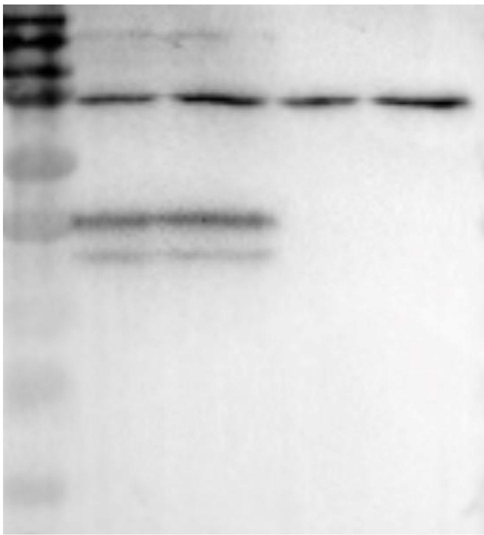

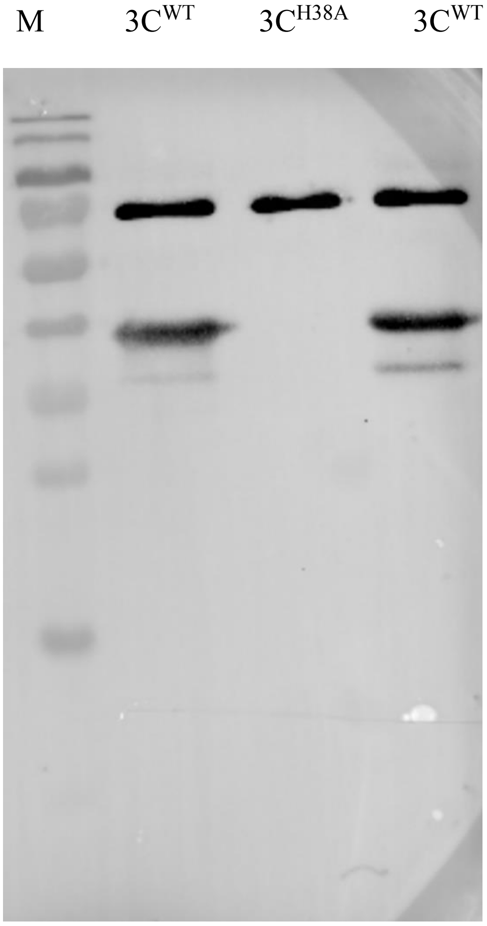

(b) Anti-PABP 70kDa 38kDa 36kDa

Figure 4. The cleavage of PABP by the DHAV 3C protease. (a) The cleavage of PABP by the DHAV 3C protease in uninfected DEF cells. After incubation with 3C protease, two cleavage products with the apparent molecular mass of 37 kDa reacted with the anti-PABP antiserum. In contrast, these two bands were not detected in the control group without 3C protease. These results demonstrated that 3C protease was sufficient to generate PABP cleavage proteins without assistance from other viral proteins. (b) The cleavage of recombinant PABP by DHAV 3C protease *in vitro*. The recombinant H38A mutant of 3C was used as a negative control. Two truncated fragments of ~36 kDa and ~38 kDa were detected specifically with anti-PABP serum, while only full-length PABP was observed in the control group.

(a)

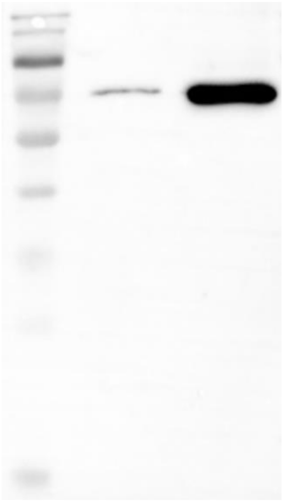

Anti-PABP 70kDa

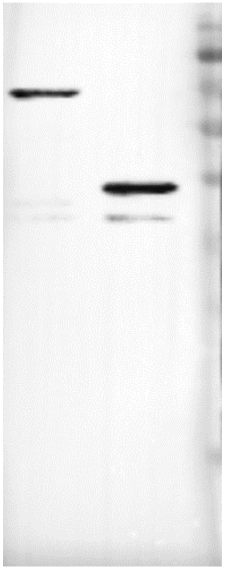

Anti-Flag 70kDa 38kDa

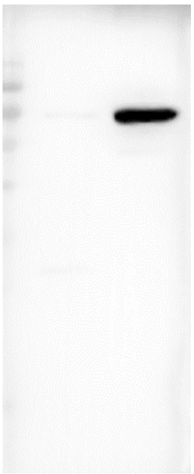

Anti-HA 70kDa 30kDa

(b)

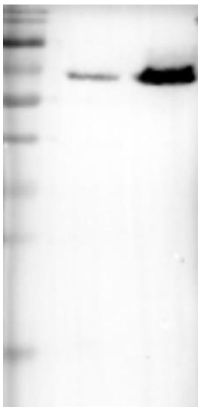

Anti-PABP 70kDa

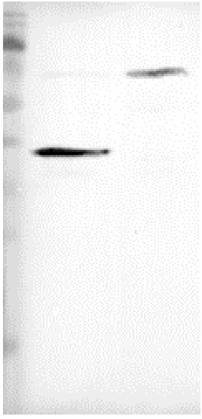

Anti-Flag 70kDa 38kDa

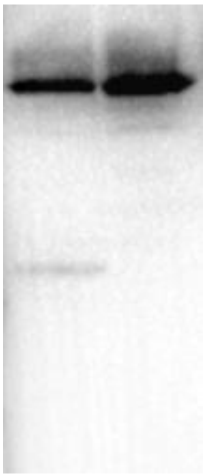

Anti-HA 70kDa 30kDa

(c)

Anti-PABP 70kDa

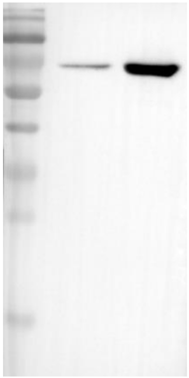

Anti-Flag 70kDa 38kDa

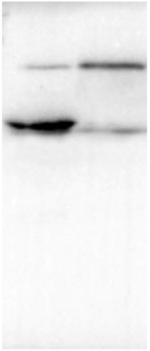

Anti-HA 70kDa 30kDa

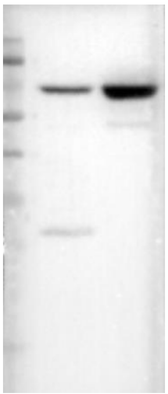

(d)

Anti-PABP 70kDa

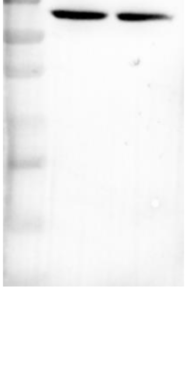

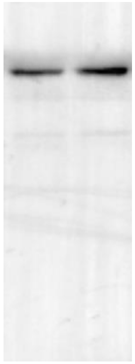

Anti-Flag 70kDa

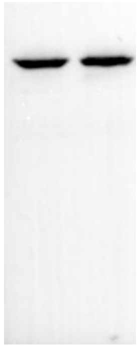

Anti-HA 70kDa

Figure 5. The cleavage of recombinant PABP and its mutants by 3C protease *in vitro*. (a) Recombinant PABP with Flag at N-terminal and HA at C-terminal was cleaved by DHAV 3C protease. (b) Recombinant of DHAV 3C protease mediated PABP mutant (G368N) cleavage. (c) Recombinant of DHAV 3C protease mediated PABP mutant (Q341A) cleavage. (d) Recombinant of DHAV 3C protease mediated PABP mutant (Q367N) cleavage. Full-length PABP was detected by anti-PABP, anti-Flag and anti-HA serum with an apparent molecular mass of approximately ~70 kDa in a 12.5% SDS PAGE gel. Anti-Flag visualized PABP derivative with N-terminal and anti-HA visualized PABP derivative with C-terminal. 3C protease reduced full-length PABP and two mutants (Q341 and G368N) level and resulted in the accumulation of a 38 kDa N-terminal and 30 kDa C-terminal PABP cleavage products, which suggested 3C protease could mediate the cleavage of PABP and mutants (Q341 and G368N). The cleavage products and the decrease of full-length PABP Q367 were not observed.

(c)

PABP siRNA Control siRNA

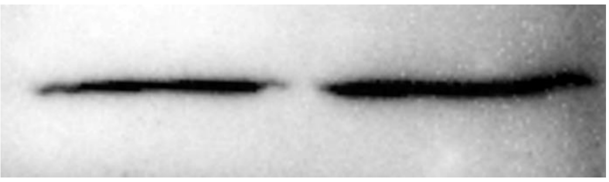

PABP 70kDa

PABP siRNA Control siRNA

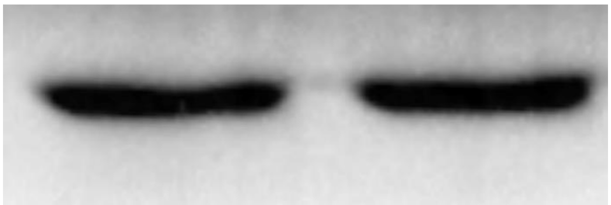

β-actin 42kDa

Figure 6. Amount of viral RNA in siRNA-mediated PABP knockdown cells.

(c)The PABP protein level in the same batch of cells was determined by immunoblotting. Cell lysates were detected with anti-PABP and anti-β-actin antibodies.

(c)

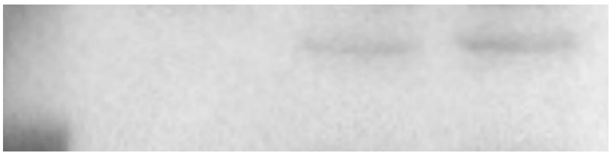

Anti-Flag 70kDa

(d)

Anti-Flag 38kDa 30kDa 70kDa pCAGGS-NTD pCAGGS-CTD pCAGGS-PABP

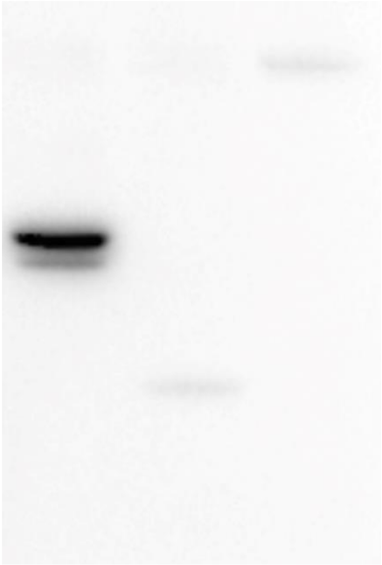

Figure 7. Amount of viral RNA in DEF cells overexpressing WT PABP, the Q367N cleavage-resistant variant and truncated PABP. (c) The cell lysates in the same batch of cells expressing WT PABP and the Q367N cleavage-resistant variant were analysed by immunoblotting. Cell lysates were detected with anti-Flag antibody. (d) The lysates of cells expressing NTD of PABP and CTD of PABP were analysed by immunoblotting. Cell lysates
